# High-Throughput Genome Editing and Phenotyping Facilitated by High Resolution Melting Curve Analysis

**DOI:** 10.1371/journal.pone.0114632

**Published:** 2014-12-11

**Authors:** Holly R. Thomas, Stefanie M. Percival, Bradley K. Yoder, John M. Parant

**Affiliations:** 1 Department of Pharmacology and Toxicology, University of Alabama at Birmingham, Birmingham, Alabama, United States of America; 2 Department of Cellular, Developmental, and Integrated Biology, University of Alabama at Birmingham, Birmingham, Alabama, United States of America; Oxford Brookes University, United Kingdom

## Abstract

With the goal to generate and characterize the phenotypes of null alleles in all genes within an organism and the recent advances in custom nucleases, genome editing limitations have moved from mutation generation to mutation detection. We previously demonstrated that High Resolution Melting (HRM) analysis is a rapid and efficient means of genotyping known zebrafish mutants. Here we establish optimized conditions for HRM based detection of novel mutant alleles. Using these conditions, we demonstrate that HRM is highly efficient at mutation detection across multiple genome editing platforms (ZFNs, TALENs, and CRISPRs); we observed nuclease generated HRM positive targeting in 1 of 6 (16%) open pool derived ZFNs, 14 of 23 (60%) TALENs, and 58 of 77 (75%) CRISPR nucleases. Successful targeting, based on HRM of G0 embryos correlates well with successful germline transmission (46 of 47 nucleases); yet, surprisingly mutations in the somatic tail DNA weakly correlate with mutations in the germline F1 progeny DNA. This suggests that analysis of G0 tail DNA is a good indicator of the efficiency of the nuclease, but not necessarily a good indicator of germline alleles that will be present in the F1s. However, we demonstrate that small amplicon HRM curve profiles of F1 progeny DNA can be used to differentiate between specific mutant alleles, facilitating rare allele identification and isolation; and that HRM is a powerful technique for screening possible off-target mutations that may be generated by the nucleases. Our data suggest that micro-homology based alternative NHEJ repair is primarily utilized in the generation of CRISPR mutant alleles and allows us to predict likelihood of generating a null allele. Lastly, we demonstrate that HRM can be used to quickly distinguish genotype-phenotype correlations within F1 embryos derived from G0 intercrosses. Together these data indicate that custom nucleases, in conjunction with the ease and speed of HRM, will facilitate future high-throughput mutation generation and analysis needed to establish mutants in all genes of an organism.

## Introduction

Manipulation of the genome in cells and in model organisms has become mainstream in biomedical research. While the mouse has prospered from advances in ES cell technology, mutant generation has been limited/more cumbersome in most other organisms. The ultimate goal is to generate mutants in all genes or in combinations of genes, and characterize the associated phenotypes; however even with ES cell technology, this is limiting. High-throughput tilling approaches to generate large collections of null alleles have alleviated this in many organisms, such as the zebrafish, C. elegans, and drosophila [1]–[7]; however, due to cost/benefit limitations, knockouts in all genes are not attainable. For example, recent efforts by the Sanger Center to obtain null alleles in zebrafish have led to mutants in approximately 50% of the known genes; yet, they have approached the saturation limit in that they are identifying more null alleles in genes where mutants have already been identified and fewer new alleles in additional genes. Genome editing in non-mouse organisms has historically been tedious and has thus been the limiting step in the utility of these model systems. Recent advances in genome editing through ZFNs (Zinc-finger nucleases) [8]–[11], TALENs (transcription-activator–like effector nucleases) [12]–[15], or CRISPR (clustered regularly interspaced short palindromic repeats) nucleases [16]–[25], has opened up opportunities in many organisms and cell lines to genome manipulation and high-throughput mutation generation. Therefore, the major limitation is now developing efficient and cost effective high-throughput strategies to detect mutants caused by these nucleases.

There are a number of techniques used for detection of genome editing derived mutations. Initially, approaches utilized the destruction of a restriction enzyme site [10], [18], [21]. However, assaying for lack of a restriction site in a PCR product is complicated by incomplete digestions and restricts the number of available target sites. The most prevalent enzymatic screen utilizes the Surveyor or T7 endonuclease mutation detection system which specifically cleaves heteroduplexed PCR products derived from newly generated heterozygous mutations [16], [18]. While reliable, these assays are tedious and time consuming. Alternatively, some labs have utilized straight sequencing of cloned PCR products [12], [17], [20]. While this approach is very informative, it becomes cost prohibitive for high-throughput screening.

**Figure 1:**
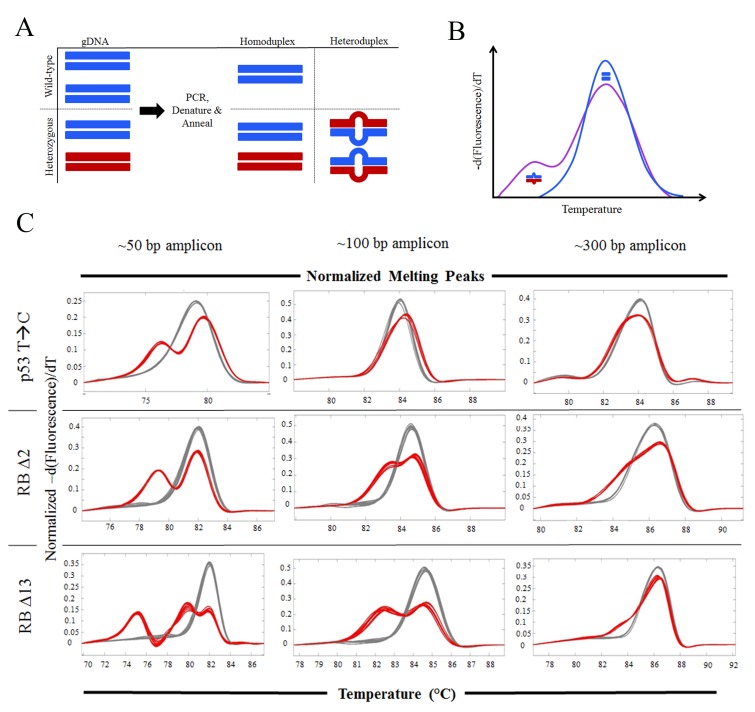
100 bp amplicons are optimal for HRM detection of nuclease induced small indels. A&B) General depiction of HRM analysis. A homozygous wild-type sample (double blue lines in A) will only produce one species following PCR, which has a specific HRM curve (blue curve in B). A heterozygous sample with a novel mutation (double red lines in A) will produce 2 homoduplex species following PCR (double blue and double red lines in A), which each have a unique HRM curve that can be separated if the T_m_ difference are great enough. Importantly for novel mutation detection, if the PCR samples are heated to 95°C and then rapidly cooled, heteroduplex products (red over blue and blue over red in A) will also be generated, which due to decreased complementarity (represented by bubble in lines in A) will have a much lower T_m_ and therefore a curve profile represented to the left of the homoduplex (see blue over red in B). C) HRM curves, of wild-type (grey curves) and either p53 (T to C missense mutation), RBΔ2, or RBΔ13 heterozygous genomic DNA samples (red curves), generated from 3 different sized PCR amplicons that surround the mutation.

To help advance cost-effective, high-throughput methodologies for mutation detection, we applied High Resolution Melting (HRM) analysis [26] for rapid and efficient identification of nuclease derived mutations. HRM is a fluorescence based technique that measures the amount of double stranded DNA at different temperatures, thereby revealing the T_m_ of a particular PCR product. While a homoduplex product generated from a homozygous DNA sample will have a particular T_m_, a heteroduplex product generated from a heterozygous individual will have an additional T_m_, generally a much lower T_m_ signature (Fig. 1A & B). It is this heteroduplex signature that expedites identification of mutant alleles. The HRM protocol requires no additional manipulations (no digestions or agarose gels) after the PCR, just a 5 minute HRM scan and in silico analysis.

To facilitate the use of HRM in high-throughput mutation detection and rapid phenotype analysis in G0 zebrafish, we established the optimal parameters and workflow for reliable detection of heterozygous alleles. We demonstrate that HRM can detect chimerism as low as ∼5% in G0 animals, and we validate that HRM is extremely efficient at mutation detection in ZFN, TALEN and CRISPR genome editing systems. Our analysis demonstrates that somatic mutations from G0 tail DNA weakly correlate with germline mutations; yet, small amplicon HRM can distinguish between individual alleles in complex backgrounds facilitating desired mutation isolation. Utilizing HRM, we determined that injection-based CRISPR genome editing produces relatively few off-target mutations; and based on HRM curve profiles, we have been able to make genotype-phenotype correlations between mutant embryos derived from G0 intercrosses, rapidly accelerating the pace of mutant phenotyping.

## Results

### Amplicon size impacts the mutation sensitivity of HRM detection

For HRM-based genotyping of unknown mutant alleles, we first had to determine the optimal amplicon size needed to accurately and reliably detect known mutations. We focused on generating the smallest amplicon possible to obtain the greatest T_m_ change between the different homoduplexes and heteroduplex PCR products. While the small amplicon will allow for greatest resolution of T_m_ differences it has a limitation in that small amplicons may not detect larger alterations (i.e. large deletions). On the converse, larger amplicons are more likely to encompass all possible mutant alleles but suffer from poor resolution of T_m_ differences. Therefore, to test the impact of amplicon size on mutation detection, we tested 50 bp (base pair), 100 bp and a 300 bp amplicon on three different mutations (heterozygous p53 mutant I166T (T>C) point mutation, heterozygous RB (retinoblastoma 1) 2 bp deletion, and heterozygous RB 13 bp deletion) designed such that the mutation was in the middle of the amplicon. For the p53 T→C point mutation, HRM could only distinguish the mutant allele with the 50 bp amplicon (Fig. 1C). However, for the RB Δ2 and Δ13 mutations, both could easily be detected utilizing the 50 or 100 bp amplicon (Fig. 1C). HRM heteroduplexes could also be detected for Δ2 and Δ13 using the 300 bp amplicon; but the heteroduplex signal is less pronounced (Fig 1C).

These data indicate that the 50 bp amplicon is best for detecting the mutations; however there is only 12–14 bp between the primers in a 50 bp amplicon, and the published mutations generated from TALENs or CRISPRs average around 10 nucleotides [17], suggesting that many of these alleles will not be detected by the 50 bp amplicon. Therefore, the ideal amplicon size, while maintaining good HRM resolution is the 100 bp amplicon. Note that for most knockout strategies, a frameshift is the desired outcome (rather than a point mutation); therefore, the lower resolution of the 100 bp amplicon compared to the 50 bp amplicon for point mutations will not be a hindrance. The second observation made from this data is that larger deletions or alterations (i.e Δ13 vs. Δ2) are easier to detect with all amplicon sizes. Also note that the large deletion (Δ13) heterozygous HRM produced two homoduplex peaks and one heteroduplex peak (far left curve; Fig. 1C) with the 50 bp amplicon. The second distinct homoduplex peak will be useful in future experiments in which homozygous Δ13 will need to be genotyped.

### HRM is highly efficient at detecting low abundant alleles

**Figure 2:**
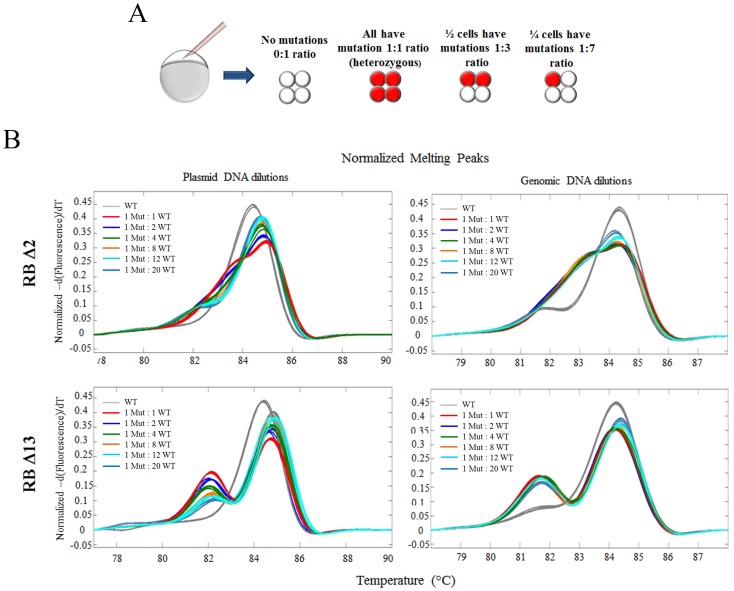
HRM can efficiently detect low level chimeric mutations. A) Depicts different degrees of chimerism (amount red colored cells) that could occur due to custom nuclease injection. B) HRM curves of TA cloned 300 bp amplicons (100 ng/ul) or genomic DNA (100 ng/ul) of wild-type (grey curves) and either RBΔ2 or RBΔ13 mutations at different allele ratios with wild-type DNA.

While the previous experiments were performed on heterozygous genomic DNA, the DNA isolated from G0 embryos is often derived from chimeric animals in which the percent chimerism depends on when during development the genomic alteration occurred (Fig. 2A). To address the detection limits of HRM for chimerism of different alleles, we tested the dilution of RB Δ2 and RB Δ13 plasmid cloned genomic DNA and true genomic DNA isolated from wild-type and heterozygous mutants. In these experiments, we diluted the plasmid or genomic DNA samples with wild-type plasmid or genomic DNA (all concentrated at 100 ng/ul) such that different allele frequencies could be examined. For the RB Δ2 as well as RB Δ13, we readily identify the mutant allele down to 1∶20 (mutant allele: wild-type alleles) dilutions, equivalent to 1 mutant alleles of a total 21 alleles, or down to 4.7% heterozygous chimerism (Fig. 2B). Interestingly, with plasmid DNA the decrease in amplitude of the heteroduplex with dilution was more apparent than with genomic DNA. This most likely reflects that there is an off-target amplicon only present with genomic DNA reactions (small curve to the left of homoduplex curve in wild-type sample). Ideally optimization of primers for the reaction would remove this off-target amplicon, but importantly this represents a realistic situation and these data suggest that mutations can be detected efficiently from a 4.7% or greater single allele chimeric animal.

### Multiple alleles within the same animal suppress the amplitude of heteroduplex peaks

**Figure 3:**
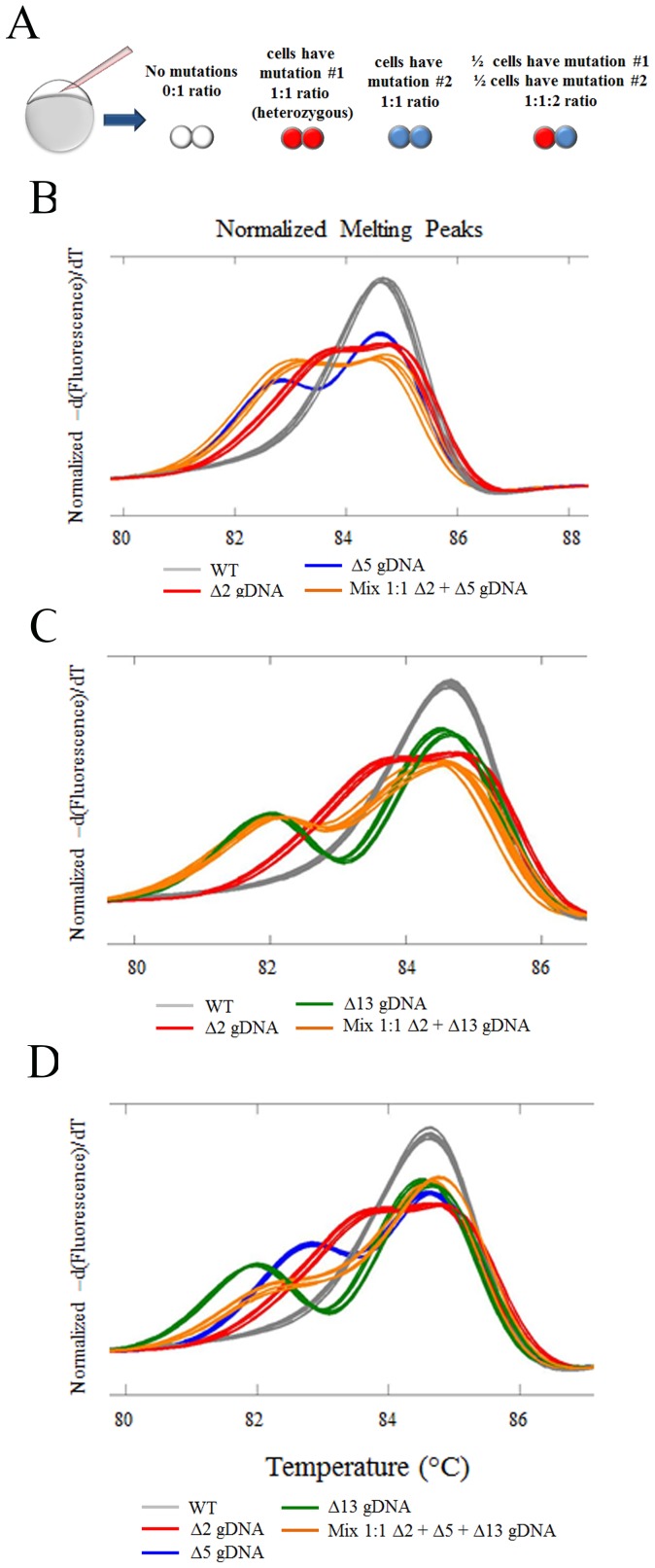
HRM can efficiently detect compound mutations. A) Depicts compound mutation (different colored cells) in a chimeric animal that could occur due to custom nuclease injection. B) HRM curves of RBΔ2, RBΔ5 and a 1∶1 mix of RBΔ2 + RBΔ5 genomic DNA; C) HRM curves of RBΔ2, RBΔ13 and a 1∶1 mix of RBΔ2 + RBΔ13 genomic DNA; D) HRM curves of RBΔ2, RBΔ5, RBΔ13 and a 1∶1∶1 mix of RBΔ2 + RBΔ5 + RBΔ13 genomic DNA.

Often custom nucleases will induce multiple targeted alleles within the same animal (Fig. 3A). In order to determine the impact that multiple alleles have on the HRM curve profile, we mixed genomic DNA from different alleles such that a 1∶1 mutant allele/s to wild-type allele ratio was maintained. When we mixed the RB Δ2 with the RB Δ5 alleles, we observed that the amplitude of the HRM curve was between that of the Δ2 and Δ5, while the left side of the shoulder followed the Δ5 heteroduplex curve (Fig. 3B). For the Δ2 and Δ13 mix, again the amplitude of the heteroduplex in the mixed HRM curve was between that of the Δ2 and the Δ13, while the left shoulder followed the left shoulder of the Δ13 heteroduplex (Fig. 3C). These data suggest the following rules when 2 different alleles are present: 1) that multiple alleles suppress the amplitude of the allele specific heteroduplex peak; and 2) that the left most shoulder follows the heteroduplex of the allele with the greatest T_m_ change.

We also analyzed the effects of mixing 3 alleles (Fig. 3D): Δ2, Δ5 and Δ13. From this mixture, we clearly detect the heteroduplex, however the curve did not follow the previous rules and gave the appearance of a less robust heteroduplex. This suggests that low amplitude heteroduplexes may indicate a multiallelic chimeric animal.

### HRM positive G0s are highly correlative with germline transmission

**Figure 4:**
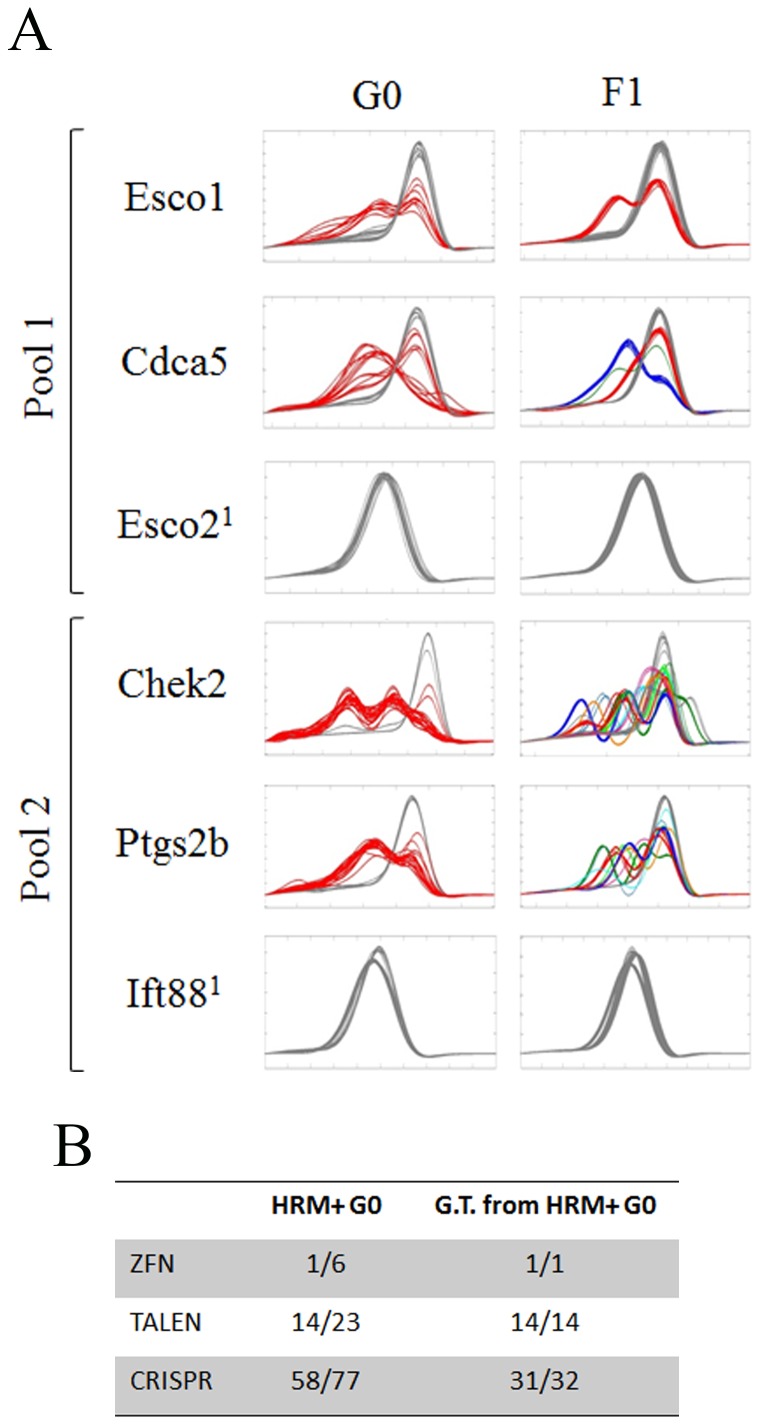
Nucleases cleavage in G0 embryo strongly correlates with germline transmission. A) HRM analysis of genomic DNA from G0 injected embryos (n = 24) and subsequent F1 progeny (n = 48) for 6 CRISPR targeted genes. Esco1, CDCa5, & Esco2^1^ (^1^ denotes a guide species that targets exon 6) were injected as pooled guide RNA (pool 1); CHEK2, Ptgs2b and IFT88^1^ (^1^ denotes the guide that targets intron 15) were injected as pooled guides (pool 2). B) Summary of HRM analysis of ZFN, TALEN, and CRISPR injected G0 and subsequent germline F1's. HRM positive G0 represent the number of different nuclease, within each category, that when injected into embryos produce at least 1 HRM positive embryo amongst 24 injected embryos; germline transmission (G.T.) from HRM+ G0 represents the number of nucleases, within each category, that were positive for HRM in injected embryos (G0) that also produce F1 progeny that contain HRM positive mutations.

One limitation for high-throughput phenotypic analysis of mutants in zebrafish is the relatively long generation time. Thus, it becomes important to know if the designed nucleases will generate a germ line transmissible mutation prior to raising G0 animals. We performed HRM to determine if nucleases that produce G0 embryos positive by HRM analysis also yield germline transmitted alleles in the F1s. For this initial analysis we injected two pools of three CRISPR guides (Esco1, Cdca5 & Esco2^1^ in one pool and CHEK2, PTGS2b, & IFT88^1^ in the second pool) with Cas9 mRNA into one cell embryos and then generated genomic DNA from 24 hpf (hours post fertilization) embryos. From this G0 genomic DNA, we determined that the Esco1^1^, Cdca5, CHEK2 and PTGS2b CRISPRs generated HRM positive curves while the Esco2^1^ and IFT88^1^ CRISPRs did not (Fig. 4A). Next we raised the injected G0 embryos, and bred them to wild-type AB fish to generate F1 progeny. We then determined that F1 progeny were HRM positive for the 4 loci identified in the G0 but not for the 2 loci that were HRM negative in the G0 embryos (Fig. 4A). This indicates that nucleases that are HRM positive in G0 embryos often result in HRM positive F1 embryos. In fact, a total of 46 of the 47 HRM positive G0 nucleases that we have tested thus far have produced germline alleles (Fig. 4B). Of note, the one that did not produce HRM positive germline mutations was a CRISPR guide for which we only obtained a few G0s. Toward the goal of generating of null alleles, 58% of TALEN derived and 64% of CRISPR derived F1 alleles were out of frame mutations.

### HRM is highly efficient at mutation detection

To test the efficiency of our HRM detection, we isolated genomic DNA from embryonic offspring (F1s) of G0 animals derived from TALEN or CRISPR injected embryos crossed to AB wild-type animals. For each clutch derived from a single G0 animal, genomic DNA from 24–48 F1 embryos was generated. HRM was performed and the percentage HRM positive (+) was determined (Table 1 and 2). In all cases, HRM + embryos were observed. To determine if our HRM + embryos truly contain nuclease induced mutations, 300 bp PCR products were generated and sequenced. From both TALEN and CRISPR derived animals, all HRM+ embryos contained novel mutations (Table 1 and 2). To determine if there were alleles undetected by HRM, such as point mutations or primer containing deletions, we sequenced 300 bp PCR products from 6 HRM negative embryos per F1 clutch analyzed. Of 48 HRM negative TALEN and 48 HRM negative CRISPR samples sequenced, no additional mutations were detected (Table 1 and 2). These data indicate that our HRM approach is highly efficient and accurate at mutation detection.

**Table 1:** HRM is highly efficient at detecting TALEN derived mutations.

G0:	% F1 HRM+ (total #)	% HRM+ with a sequence mutation	Alleles identified	% HRM- with a sequence mutation
PUMA G0#1	33% (n = 36)	100% (n = 6)	Δ2, Δ5	0% (n = 6)
PUMA G0#2	16% (n = 38)	100% (n = 6)	Δ8	0% (n = 6)
PUMA G0#3	26% (n = 42)	100% (n = 6)	Δ11+6, Δ9^1^	0% (n = 6)
PUMA G0#4	42% (n = 24)	100% (n = 6)	Δ9^2^	0% (n = 6)
MDM4 G0#1	28% (n = 36)	100% (n = 6)	Δ4+7	0% (n = 6)
MDM4 G0#2	42% (n = 36)	100% (n = 6)	Δ9, Δ3+10	0% (n = 6)
Cyclin G1 G0#1	15% (n = 48)	100% (n = 6)	Δ4	0% (n = 6)
Cyclin G1 G0#2	17% (n = 36)	100% (n = 6)	Δ10	0% (n = 6)

HRM analysis was performed on F1 progeny derived from G0 (from 3 different TALEN targeted genes) crossed to wild-type AB strain. Percentage F1 that are HRM positive represents the percentage of HRM positive embryo per the total number of embryos analyzed; percent HRM positive with sequence mutation represent the frequency that HRM+ embryos (6 embryos per G0 were analyzed) also contain a sequence mutation; Alleles identified represent the exact mutation identified through sequencing; percent HRM negative with a sequence mutation represents the percentage of HRM negative samples (6 per G0 were analyzed) that when sequenced with a 300 bp amplicon contained a mutation.

**Table 2:** HRM is highly efficient at detecting CRISPR derived mutations.

G0	% F1 HRM+ (total #)	% HRM+ with a sequence mutation	Alleles identified	% HRM- with a sequence mutation
BARD G0#1	77% (n = 30)	100% (n = 6)	Δ2^1^ and Δ5	0% (n = 6)
BARD G0#2	75% (n = 24)	100% (n = 6)	Δ2^1^ and Δ16	0% (n = 6)
Bub1bb G0#1	31% (n = 36)	100% (n = 6)	Δ18	0% (n = 6)
Bub1bb G0#2	50% (n = 18)	100% (n = 6)	Δ4, Δ7, and Δ9+24	0% (n = 6)
p107 G0#1	80% (n = 30)	100% (n = 6)	Δ3^1^, Δ6^1^, +5 and Δ14+3	0% (n = 6)
p107 G0#2	71% (n = 24)	100% (n = 6)	Δ3^1^, Δ6^1^ and +17	0% (n = 6)
ATAD5b G0 #1	63% (n = 24)	100% (n = 6)	Δ1, Δ5^1^, and Δ7	0% (n = 6)
ATAD5b G0 #2	23% (n = 30)	100% (n = 6)	Δ5^1^	0% (n = 6)

HRM analysis was performed on F1 progeny derived from G0 (from 4 different CRISPR targeted genes) crossed to wild-type AB strain. Percentage F1 that are HRM positive represents the percentage of HRM positive embryo per the total number of embryos analyzed; percent HRM positive with sequence mutation represent the frequency that HRM+ embryos (6 embryos per G0 were analyzed) also contain a sequence mutation; Alleles identified represent the exact mutation identified through sequencing; percent HRM negative with a sequence mutation represents the percentage of HRM negative samples (6 per G0 were analyzed) that when sequenced with a 300 bp amplicon contained a mutation.

### Small amplicon HRM allows for specific allele identification

**Figure 5:**
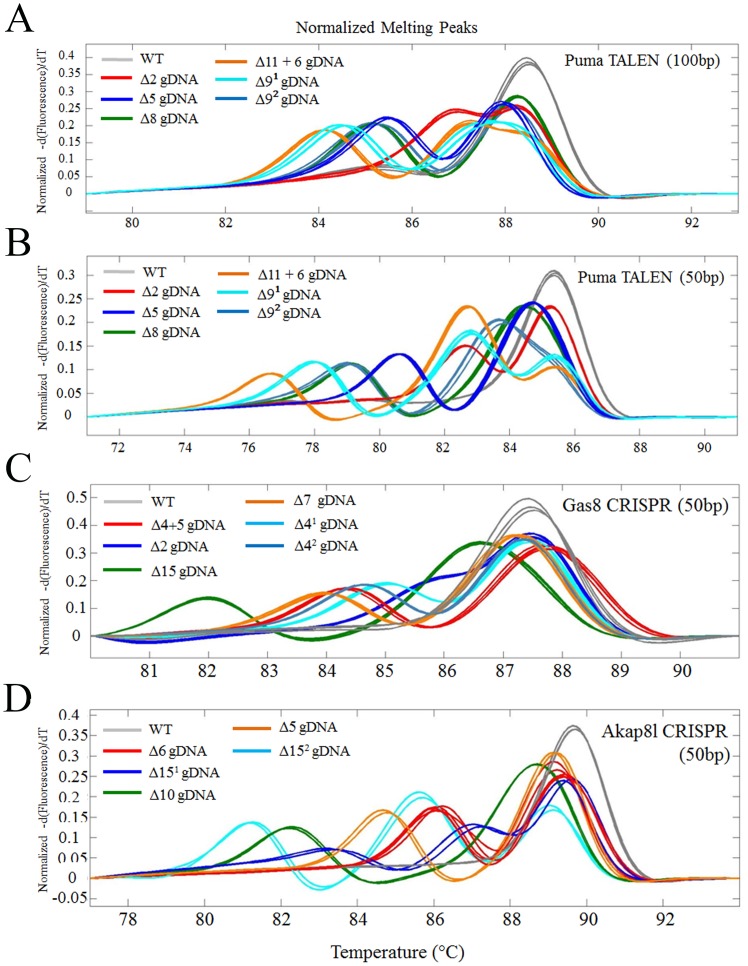
Small amplicon HRM can distinguish distinct mutant alleles. A) 100 bp amplicon of different PUMA alleles in F1 progeny of TALEN derived G0's fish; each curve denotes a different mutation. B) HRM of the same genomic DNA as in A) with a 50 bp amplicon across the PUMA target site enhanced curve separation. Identification of 5 different HRM curves in F1 progeny genomic DNA from a (C) Gas8 CRISPR G0'sor (D) AKAP8I CRISPR G0's. Subsequent sequencing revealed that each curve is the result of a unique mutation.

While analyzing the data from Table 1 and 2, we often observed distinct HRM curve profiles amongst different F1 animals. Following sequencing, we found that specific profiles are indicative of specific mutations. For example, we observed that each distinct mutation identified from the PUMA TALEN had a specific HRM curve profile (Fig. 5A). Further we demonstrate that a 50 bp amplicon provides a more distinct separation of HRM profiles (Fig. 5B). This HRM profile based allele segregation could alleviate the need to sequence many F1 progeny for additional alleles, and provide a means to identify a particular desirable allele from F1 fish. To determine the efficiency of allele segregation using the 50 bp amplicon HRM profiles, we performed HRM on F1 embryos derived from multiple Gas8 and AKAP8I targeted G0 fish, and identified 5 distinct profiles for each. We then sequenced DNA from 3 individuals with each specific profile (15/gene). In all cases (30 of 30) the HRM profile has a perfect correlation with the exact mutation discovered (Fig. 5C&D). These data suggest that HRM can facilitate novel allele identification, as well as future allele differentiation. These data also corroborate with Fig. 1, in that larger disruptions or more complex alterations result in a more pronounced T_m_ change in the heteroduplex curve.

### The mutations and allele frequencies in somatic (tail) DNA do not correlate with the mutations and frequencies identified in DNA from germline progeny

For high-throughput null mutation generation, we envisioned that identification of a G0 animal with a specific allele mutation in the soma (tail DNA) might facilitate identification of animals with a desired allele. To test this, we generated genomic DNA from tail clippings of 8 different G0 fish that had been injected with an Esco2^2^ CRISPR. From each tail, a 300 bp amplicon was generated, cloned and mutations were sequenced. We also isolated genomic DNA from 24 F1 progeny, for which we identified the mutation produced. Interestingly, from these data, we observed a lower number of alleles in the germline than in the soma (Table 3). In most cases the allele found in the germline also occurred in the soma; suggesting these mutational events must have occurred before primordial germ cell specification. G0#4 and #6 were the exception, in which the mutant alleles Δ1+7 and Δ21, respectively, were detected in the germline but not detected in the tail DNA. The 50% frequency of the unique germline Δ21 in #6 suggests that the event must have happened early, but uniquely in the primordial germ cells. These data suggest that F1 mutation analysis is more valid as far as germline contribution than somatic analysis.

**Table 3:** Frequency and mutation do not always correlate from tail to germline.

G0 number	Alleles detected in tail DNA (frequency)	Alleles detected in F1 progeny (frequency)
1	**D7^1^ (7/36)**	**D7^1^ (3/24)**
1	D21^1^ (1/36)	
2	**D7^1^ (1/32)**	**D7^1^ (3/24)**
2	D7^2^ (3/32)	D7^2^ (1/24)
2	D1^1^ (3/32)	
2	D14+5^1^ (2/32)	
3	**D7^1^ (7/30)**	**D7^1^ (3/24)**
3	D7^2^ (6/30)	
3	D12^1^ (2/30)	
3	D12^2^ (2/30)	
3	D12+6^1^ (1/30)	
4	D7^2^ (7/41)	D1+7^1^ (5/24)
4	D8^1^ (3/41)	
4	D15^1^ (1/41)	
5	D7^2^ (7/32)	**D7^1^ (4/24)**
5	+51 (1/32)	D7^2^ (3/24)
6	**D7^1^ (1/36)**	D7^2^ (3/24)
6	D7^2^ (1/36)	D21^1^ (12/24)
6	D9^1^ (4/36)	
6	D12^1^ (2/36)	
6	D17^1^ (1/36)	
6	D8^1^ (1/36)	
7	D7^2^ (9/35)	D7^2^ (11/24)
7	D12^1^ (2/35)	D12^1^ (3/24)
8	**D7^1^ (13/34)**	**D7^1^ (8/24)**
8	D7^2^ (2/34)	+2^1^ (2/24)
8	D12^1^ (5/34)	

Table displays mutations and frequency of mutations derived from TA cloned 300 bp amplicons of G0 tail and F1 progeny genomic DNA.

### Micro-homology mediated end joining is the preferential means of repairing CRISPR induced breaks

**Figure 6:**
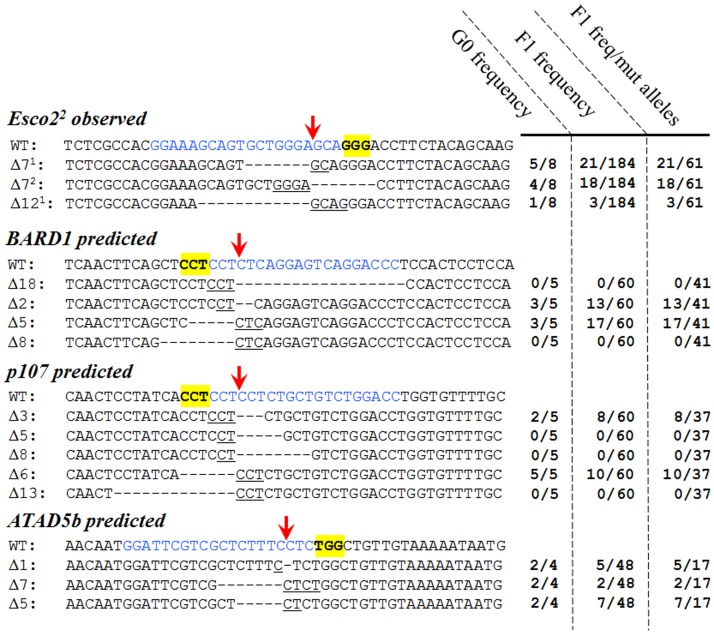
Micro-homology directed repair is the prevalent repair mechanism of CAS9 induced cleavage. From sequence data obtained in Table 3 three dominant alleles were observed (Δ7^1^, Δ7^2^, and Δ12^1^). Alignment of wild-type and mutant sequences (yellow highlight denotes PAM, blue denotes target sequence, and red arrow denotes cleavage site) are displayed, and potential micro-homologies are underlined. G0 frequency represents how many of the G0 analyzed produced at least one embryo with this allele; F1 frequency represent how often we observed this allele in total F1 analyzed; and F1 Frequency/mutant allele represents how often we observed this allele amongst all mutant allele discovered. For BARD1, p107, and Atad5b predicted mutations are shown based on underlined micro-homologies. Actual observed frequencies are displayed to right.

Through the analysis of the Esco2^2^ mutant alleles (Table 3) we observed a preference for generating particular alleles. The same Esco2 Δ7^1^ allele was derived from 5 of 8 different G0 animals (soma and progeny); while the other Δ7^2^ was identified in 7 of 8 G0 based on tail DNA and 4 of 8 G0's based on F1 germline contribution. Among the total mutant alleles identified from the 8 G0, the two Δ7 alleles made up 39 of 61 alleles (Fig. 6). In addition, in the soma Δ12^1^ also occurred often, in 4 of 8 G0's (tail), while it only occurred in 1 of 8 of the G0's progeny (F1). When we align the sequence of these preferential deletions, we observe that they often occur between micro-homology regions (2 or 4 nucleotides on the PAM (Protospacer Adjacent Motif) side of cleavage, and 4 nucleotides on the other side of cleavage; Fig. 6) on either side of the Cas9 directed break (3 nucleotides 5′ of PAM). This suggests that alternative non-homologous end joining must be the preferential mechanism of DNA repair [27]–[29]. Further, this indicates that we can potentially predict the alleles acquired from a specific guide.

To evaluate this observation, we made predictions of the prevalent allele in our BARD1, p107 and Atad5b gene targeting, based on target sequence and the following rules: that the CAS9 cuts 3 nucleotides upstream of the PAM sequence; and larger micro-homologies (3–5 nucleotides) are preferential to smaller homologies (1–2 nucleotides) (Fig. 6). Using the sequence data from Table 2 and additional sequence data, we found the predictions to be accurate. In the case of BARD1, the predicted prevalent alleles would be a Δ2, Δ5, Δ8 and Δ18 (based on 2–3 nucleotide micro-homology). From 5 G0s analyzed, we observed 3 G0s that produced the Δ2, and 3 G0s produced the Δ5, but no G0s produced Δ8 or Δ18; suggesting proximity to the cut site may be preferred. For the p107 targeting, we predicted a Δ3, Δ5, Δ6, Δ8, and Δ13 (based on 2–3 nucleotide micro-homology) alleles, and observed Δ3 in 2 of 5 and Δ6 in 5 of 5 G0 progeny, but no Δ5 Δ8 or Δ13 in the G0 progeny; again suggesting a proximity bias to the cut site. For Atad5b, we predicted Δ7 (based on 4 nucleotide micro-homology), and potentially Δ1 and Δ5 (based on 1–2 nucleotide micro-homology) alleles. We observed all three alleles in progeny from 2 of the 4 G0s. Overall these predicted alleles make up a majority of the derived alleles: 30 of 41 (73%) BARD1 mutations, 18 of 37(49%) p107 mutations; and 14 of 17(82%) Atad5b mutations (Fig. 6). Together these data support the idea that micro-homology based repair is a preferred repair mechanism. These data also suggest that smaller micro-homologies, such as those used in the Atad5b Δ5 and Δ1 alleles, may be utilized if larger homologies do not exist, and suggest a preference for the nearest homology on the other side of the break. Interestingly, we did not observe the prevalence for common alleles with TALENS, but this may reflect the lower specificity of the exact cut site due to the spacer between the Fok1 enzyme and the TALEN DNA binding domain.

### HRM can be used to determine off-target mutations

**Figure 7:**
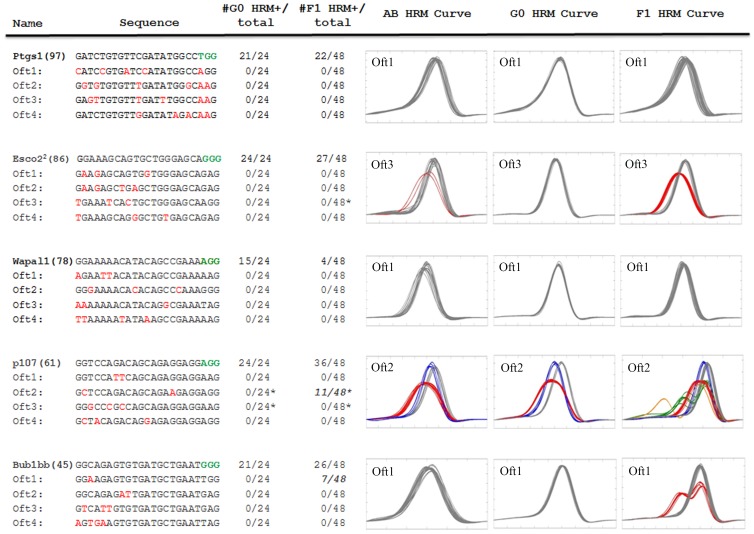
HRM analysis reveals low frequency of off-target cleavage by the CRISPR system. Using the CRISPR Design (http://crispr.mit.edu/) web tool we identified the most likely 4 off-target sites in the zebrafish genome for 5 different guides with different overall scores (in parenthesis next to gene name). The sequences are depicted with the PAM site in green and the nucleotide different from the guide sequence in red. To discern SNP's within the Oft amplicons we performed HRM analysis of genomic DNA from 24 wild-type adults. The HRM positive frequency within genomic DNA obtained from 24 G0 embryos injected with the guide/Cas9 RNAs is displayed. Also the HRM positive frequency within genomic DNA obtained from 12 F1 embryos derived from 4 different G0's is displayed. Select HRM curves that display unique features are displayed for G0 embryos as well as F1 embryos. For Ptgs1 Oft 1 is displayed to depict no off-target hits. For Esco2 Oft 3 is displayed to depict a common SNP derived from the wild-type AB. For Wapal1 Oft 1 is displayed to depict no off-target hits. For P107 Oft 2 is displayed to: 1) depict a SNP found in wild-type AB strain, G0 and F1 animals; and 2) novel CRISPR derived off-target hits in some of the F1 progeny (orange and green curves). For Bub1bb, Oft 1 is displayed to depict a low frequency off-target hit (red curve) in the F1 progeny. * in frequencies denotes that SNP curves were not considered a HRM positive.

One concern of genome editing, in particular with CRISPR genome editing, is the potential for off-target cleavage [16], [18], [25], [30], [31]. To address this, we have analyzed multiple guides using the CRISPR Design (http://crispr.mit.edu/) online tools. From this, we selected 5 guides that range in score from 97 (Ptgs1) to 45 (Bub1bb) and generated 4 off-target (Oft; the most likely 4) HRM amplicons for each of the 5 guides to determine if these sites are being targeted (Fig. 7). To exclude alternative HRM curves derived from SNP's contained within the Oft amplicon, we performed HRM analysis of 24 randomly selected AB (wild-type parental strain) adult zebrafish. In the case of Esco2^2^ Oft 3, p107 Oft 2, and P107 Oft 3 we observed distinct HRM curves that when sequenced have SNP's. Of the 20 off-targets analyzed, none were positive for HRM in 24 G0 embryos analyzed. Notice in the case of p107 Oft 2 (also p107 Oft 3, data not shown) we did observe the background SNPs (red and blue curve in Fig. 7 AB profile) in the G0 embryos (red and blue curve in Fig. 7 G0), while we did not in the Esco2^2^ Oft 3. There is the possibility that off-targets are below the limits of detection in G0 animals (<4.7% chimeric based on Fig. 2). Therefore we also isolated genomic DNA from 12 F1 embryos each from 4 different G0s (48 F1s/gene). With Esco2^2^, Ptgs1 and Wapal1 we did not observe any off target mutations in the 4 off-target sites. For p107, 3 of 4 off targets did not have alternative curves (excluding the SNP in Oft 2 and Oft 3), while Oft 2 clearly had 11/48 embryos with clear HRM positive curves (2 different curves). Sequencing of these two curves revealed a Δ15+3 mutation and a 2 base pair (bp) insertion at the CRISPR off-target cut site, consistent with a CRISPR derived mutation. For Bub1bb, which has the worst overall score, only Oft 1, which has a single nucleotide change in the target site at position 3, produced off-target hits in 7 of 48 F1 embryos. Sequencing revealed this to be a 3 bp insertion, at the CRISPR cut site. Interestingly in both off-target cases, we did not observe the off-target hit in G0 embryos, but did in the F1 progeny; suggesting that off-target analysis needs to be performed on the F1 progeny not G0 injected embryos. Furthermore, in both cases, the germline frequencies for the off-target mutation were lower than the on-target germline frequencies, suggesting that we can easily segregate these alleles apart. Together these data indicate that off target hits are low (2 of 20 targets analyzed) and preferentially target “off target” sites with NGG PAM sequences not NAG (2 of 6 with NGG verses 0 of 14 with NAG). In addition, higher scoring guides (>78) generally did not have off- target hits while lower scoring (<61) did. These data suggest that HRM can be used to identify off-target frequencies and can be used to select embryos/fish without off-target mutations or delineate phenotypes that are due to off target hits.

### Using HRM we can efficiently segregate individual alleles from pooled guide injections

To facilitate higher throughput mutation generation or rapid generation of complex mutants, we want to pool multiple guides into single injections; however this potentially produces a bottleneck in isolation of single gene mutant F1's. To determine if we can use HRM to efficiently isolate single gene mutants, we raised F1 progeny from 3 G0 animals, derived from a pooled injection of Gas8, Ptgs1, and IFT88^2^ guides, and performed HRM analysis of the 3 targets in 86 F1 adults. From this, we were able to identify 18 Gas8; 12 Ptgs1, and 14 IFT88^2^ animals that contain single gene mutations (Table 4). We also were able to obtain 3 triple heterozygous animals suggesting that pooling guides can quickly generate triple heterozygous animals. To expand this approach, we generated additional G0 animals from pooled guides, of which 4 of 4 tested have produced single allele mutant offspring as well as multi-loci heterozygotes. Overall these data suggest that HRM can efficiently isolate unique single gene mutants, or triple heterozygous mutations, if desired. Such strains would be an important resource for analyzing the contribution of complex mutants to disease states.

**Table 4:** HRM can efficiently identify single gene mutation containing F1 from pooled guide injections.

Table 4: Genotypes from G0 (Gas8;PTGS1;Ift88) x AB	G0#9 (#/total)	G0#3 (#/total)	G0#6 (#/total)
Gas8^+/M^; PTGS1^+/+^; Ift88^2+/+^	12/49	3/20	3/27
Gas8^+/M^; PTGS1^+/M^; Ift88^2+/+^	5/49	0/20	5/27
Gas8^+/M^; PTGS1^+/+^; Ift88^2+/M^	5/49	5/20	0/27
Gas8^+/M^; PTGS1^+/M^; Ift88^2+/M^	3/49	0/20	0/27
Gas8^+/+^; PTGS1^+/M^; Ift88^2+/+^	7/49	0/20	5/27
Gas8^+/+^; PTGS1^+/M^; Ift88^2+/M^	8/49	1/20	0/27
Gas8^+/+^; PTGS1^+/+^; Ift88^2+/M^	6/49	7/20	1/27
Gas8^+/+^; PTGS1^+/+^; Ift88^2+/+^	3/49	4/20	13/27

Genotypes were determined by HRM analysis of tail DNA from F1 adult fish derived from pooled guide injected G0 animals. Frequencies are denoted as number with genotype per total number of animals genotyped. Note different mutations within the same target site are considered the same for this analysis.

### GC content impacts effectiveness of guides

**Figure 8:**
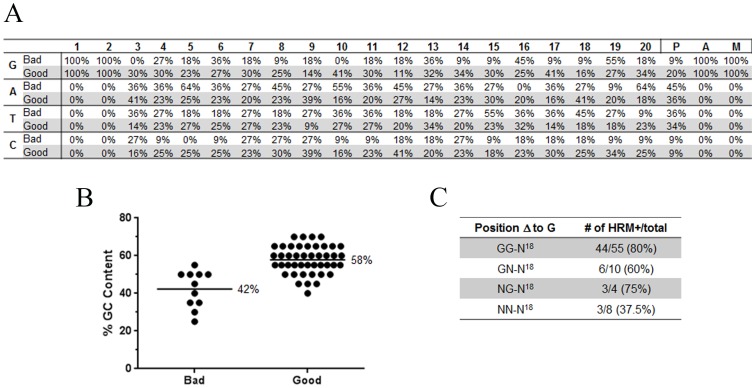
GC content impacts effectiveness of guides. A) denotes the percent G, A, T, or C content by position among all Bad or Good perfect match guides, based on if they produce an HRM positive curve in G0 embryos. B) Dot plot of GC content of BAD and GOOD guides. C) Success of guides that have either position 1 or/and 2 changed to a G (non-perfect match guides).

**Figure 9:**
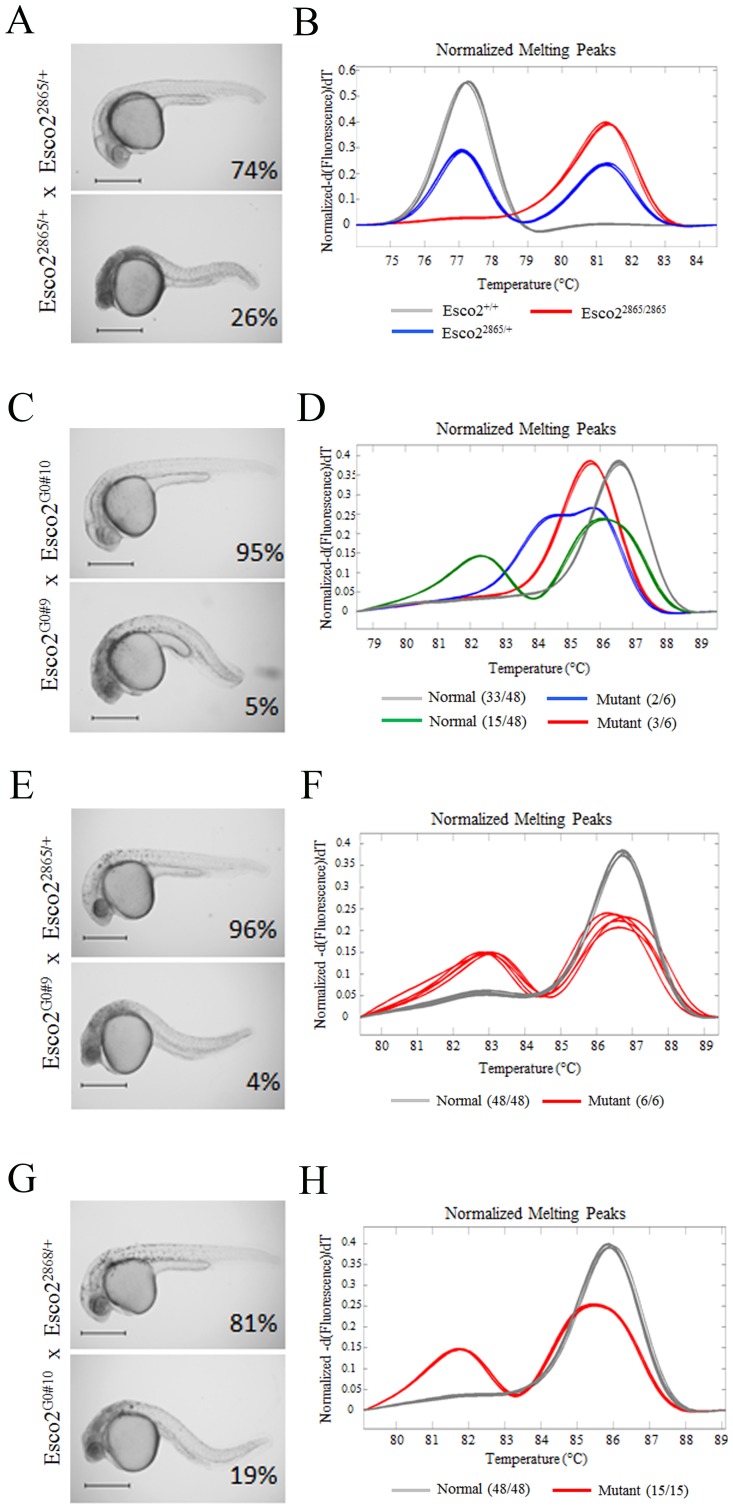
HRM established genotype-phenotype correlation within Esco2 mutant embryos from a G0 intercross. A) wild-type and mutant phenotypes with Mendelian frequencies in embryos derived from heterozygous intercross of the Esco2 retroviral insertion mutant hi2865. Note the head necrosis in the mutant embryos. B) HRM genotyping of wild-type (grey and blue curves) and mutant (red curves) embryos display perfect genotype-phenotype correlation. C) wild-type and mutant phenotypes in embryos derived from intercross of G0 Esco2 CRISPR injected fish. Note the head necrosis in mutant embryos. D) Select HRM curves of 6 mutant and 6 wild-type embryos that were subsequently sequences to reveal specific alleles result in specific curves. All Wild-type animals (beyond the 6 displayed here) make up the green and grey curves; while 5 of 6 mutant animals make up the unique red and blue curves establishing a genotype phenotype correlation. E&G) are wild-type and mutant phenotypes and frequencies of G0#9 (E) or G0#10 (G) crossed to Esco2 hi2865 heterozygous animals. F&H) are HRM curves of mutant and wild-type embryos (from E&G) that are Esco2 hi2865 heterozygous. All heterozygous curves (red) are mutants, and all grey curves are normal phenotypically.

Throughout this work we have generated 55 perfect match guides, of which 11 did not produce an HRM positive curve in G0 embryos. To evaluate if the nucleotide content of the guides at specific positions influences their effectiveness, we analyzed the composition of each of the 20 nucleotide target sequence positions and the 21^st^ position in the PAM sequence (Fig. 8A). While there were composition differences we did not observe clear trends. More striking was that guides with a higher GC content were more likely to work that lower GC content guides (Fig. 8B). Due to the need for a GG on the 5′ end of the guide to get effective transcription from the T7 promoter, we often change the last two nucleotides to GG. To evaluate if this influences the effectiveness of the guides we compared the success for different alterations based on position. While single nucleotide changes to a G at position 1 or 2 did not decrease their effectiveness (60% and 75% respectively), changes of both 1 and 2 position decreased the effectiveness down to 37%, suggesting these alteration should only be used if essential (Fig. 9C).

### Rapid phenotyping using chimeric G0 animals

In addition to high throughput generation of null mutations in all genes, preliminary assessment of homozygous phenotypes is desired. The high chimerism of G0 animals and the low off-target rate of CRISPRs should allow for phenotypic analysis in the F1 generation. To test this, we established a phenotype-genotype correlation of F1 embryos derived from Esco2^2^ guide injected G0 animals. We have previously characterized the Esco2 homozygous mutant (hi2865)[32] head necrosis phenotype occurring at normal Mendelian inheritance (1∶3) from a heterozygous intercross (Fig. 9A & B). From a pair of G0 Esco2 CRISPR injected G0 animals, we observed that 6 of 130 F1 progeny at 24 hpf had the predicted Esco2 mutant head necrosis phenotype, while the rest of the clutch was phenotypically normal (Fig. 9C). To establish that these phenotypes were dependent on being a particular genotype (potentially homozygous or compound heterozygous for a mutant allele), we performed HRM on the 6 mutant and 48 wild-type embryos. Among the 48 wild-type embryos, we only observed two HRM profiles: 1) majority (33 of 48) had a homozygous wild-type profile (Fig. 9D grey curve), suggesting the G0s were low chimeric; and 2) a heterozygous profile in 15 of 48 embryos, that resembled the previously identified Δ7^1^ or Δ7^2^ alleles (Fig. 9D green curve; Table 3). From the mutants we observed 3 curve profiles: 1) 3 embryos appeared to be homozygous for a mutant allele (lack of a heteroduplex hump, but homoduplex shifted to the left; Fig. 9D red curve); 2) 2 embryos appeared to be compound heterozygous (contained a novel heteroduplex peak; Fig. 9D blue curve); and surprisingly 1 embryo had a wild-type HRM profile (Fig. 9). Importantly the first two mutant profiles (Fig. 9D blue and red) do not appear in any of the 48 wild-type animals, suggesting these are mutant specific and make a genotype (HRM based)-phenotype correlation. PCR amplicons (300 bp) from the 6 mutants (based on phenotype), 6 heterozygous (based on HRM curve) and 3 wild-type (based on HRM curve) animals were sequenced and revealed that all wild-type HRM curve animals were +/+, all heterozygous were Δ7^2^/+, the homozygous mutants were Δ7^2^/Δ7^2^, the compound heterozygous mutants were Δ7^1^/Δ7^2^, and the wild-type HRM animal with the mutant phenotype was +/+. This phenotypically mutant/genotypically wild-type embryo could represent an unrelated bad embryo or potentially an off target mutant. To determine the mutant frequency from each G0 and to ensure the alleles induce an Esco2 mutant like phenotype, we crossed each G0 to Esco2 hi2865 heterozygous fish. (Note this genomic alteration is outside of the CRISPR target area and requires separate genotyping). For G0#9 X Esco2^2865/+^ we observed 6 of 147 embryos (4%) displayed a mutant phenotype (Fig. 9E). Since the cross was to a hi2865 heterozygous in which only half the embryos would receive the hi2865 allele, then 12 of 147 (8%) progeny should carry a #9 allele irrespective of phenotype. The percentage germline heterozygous chimerism in the G0 is 16% (2×8%; since it is heterozygous). From HRM analysis of only the embryos genotyped to be Esco2 2865 heterozygous, we observed all 6 mutants to have heterozygous Esco2 CRISPR derived Δ7 curves; and all phenotypically normal (25 of 25) embryos to have wild-type Esco2 curves (Fig. 9F); indicating these mutant phenotypes are dependent on the Δ7 allele. For G0#10, we observed a close to Mendelian frequency (15 of 78 were mutant; 19% mutant) of mutant phenotypes (Fig. 9G) suggesting this was a high chimeric animal (∼77% of germline heterozygous for null mutation.) Amongst Esco2 2865 heterozygous embryos, we observed 15 of 15 mutant embryos had a Δ7 heterozygous curve, while all (32 of 32) normal embryos had a wild-type HRM curve (Fig. 9H). Together this data corroborates the observed G0 intercross 4% mutant frequency (actually 3.4% based on genotype (5/147)), when the expected frequency from crossing a 16% chimeric heterozygous G0 to a 77% heterozygous G0 is 3.1% ((0.16×0.5)×(0.77×0.5)×100)). These data not only suggest that chimeric G0 crosses can be used for rapid phenotyping of mutants, but in conjunction with the HRM curve profiling, can easily generate genotype-phenotype correlations.

## Discussion

### Overview of genome editing

Utilizing HRM, we have successfully targeted sites with 1 of 6 (16%) open pool derived ZFNs, 14 of 23 (60%) TALENs (S1 Table), and 58 of 77 (75%) CRISPRs (S2 Table). In our hands TALENs and CRISPRs are superior in gene targeting to ZFNs. Based on our analysis, TALENs produce 1–2 alleles per G0 while CRISPRs generate up to 4 different alleles per G0. This suggests that CRISPRs may be more beneficial since 1/3 of alleles will be in-frame (Fig. 8C) and do not generate a null allele; however, in-frame deletions could generate a biological null by altering protein structure. Further, in our hands, CRISPRs are also less complicated in the time required to make the guide, the reduced need to vary injection concentration, and the ability to mix multiple guides. Of note we did observe higher targeting efficiency by HRM with the codon optimized Cas9 [18] than with the unaltered Cas9 [17] but this may also reflect in vitro RNA polymerase (T3 v. T7). CRISPRs have an overall 75% (58/77) functional guide success rate, therefore we find that mixing 3 guides to the same gene ensures high probability of identification of a null allele, as well as potential deletion alleles. We have used both ZFIT (http://zifit.partners.org/ZiFiT/)[33] and CRISPR Design (http://crispr.mit.edu/)[16] for guide design and have found no correlation with better success. Further the quality score from CRISPR Design does not correlate with the success of the guide, but does correlate with the probability of off-target sites; i.e. p107 had a low score of 61, while Bub1bb had a low score of 45, and both had one HRM positive off-target site out of the total 20 off-target sites tested (4 sites each for 5 genes). Importantly GC content above 58% correlates well with successful targeting. These observations are consistent with the recent report of greater success with GC contents above 50% [34]. Further, we have demonstrated that there is predictability on the cleavage derived alleles; micro-homology mediated repair will be utilized if such micro-homology exists on both sides of the cleavage site. While this data is exclusively from zebrafish, surveying a limited number of publications in zebrafish [34], Drosophila melanogaster [35], C. Elegans [21], [36], goat [37] and human cell lines [38] this micro-homology mediated repair is consistent through these organisms. It should be noted that if there are not clear micro-homologies nearby the cleavage site, alternative repair mechanisms are utilized and the mechanism by which insertions occurs is still unclear.

While the ideal target sequence is “GG-N^19^-GG, at times we have substituted the 5′ one or two nucleotides with G's to allow for ideal in-vitro transcription. Amongst perfect matches (GG-) we observe a 80% (44/55) success rate for any designed guide. Our data suggest that changing position 1 (NG-) has minimal effect and results in a 75% (3 of 4) success; changing position 2 only (GN-) decreases the success to 60% (6 of 10); and changing both position 1 and 2 (NN-) reduces the success to 37.5% (3 of 8). Therefore our criteria for generating null alleles are to select target sites with at maximum one mismatch at position 1 or 2. If a particular location is required, mismatches at 1 and 2 can be designed but may require multiple guides to identify an efficient one. Of guides with 2 mismatches that function, they all cleave well and therefore it is not a matter of reduced efficiency.

Our observation of disconcordance of somatic mutations with germline mutations most likely reflects the timing of cleavage and the timing of primordial germ cell (PGC) differentiation. At the 1000 cell stage there are 4 PGC. While it has been assumed that DNA cleavage happens very early (1–4 cell stage), our data suggest that some cleavage events do not occur until later developmental stages when the PGC differentiate. Further, the fact that we observed identical alleles from different G0 individuals suggest that many of the high frequency alleles may not be a derivative of an earlier nuclease cleavage but a preponderance for generation of a particular allele later in development within different cells.

As far as biallelic targeting, based on our germline contributions of TALEN-derived alleles, we rarely observed a germline transmission frequency greater than 50%, suggesting the best case scenario is that they are heterozygous; whereas with CRISPRs we often observe greater than 50% germline contribution. However, this may be biased depending on if the target gene is essential or not. In fact, with genes we know are homozygous non-viable, we often observe more in-frame mutations in G0s with greater than 50% contribution. Our data also indicates that among guides that do produce off-target cleavage, the off-target cleavage event is infrequent and less efficient compared to the target site cleavage. Further, such low frequency off-target hits can be segregated out more quickly using off-target HRM genotyping.

### Impact of HRM on genome editing

While traditionally the bottleneck has been the lack of efficient techniques for custom nuclease cleavage, the new genome editing techniques have made this step proficient and readily available. Now the major bottleneck exists in mutation detection. We demonstrate that HRM can be used to alleviate this bottleneck. HRM is extremely unrestrictive, in that as long as you can PCR across a target site, HRM can be used. HRM is rapid; from genomic DNA to mutant identification only takes 1 hour. Furthermore, once the HRM equipment is in place, HRM is low cost and can detect low level chimeric animals, which other techniques often cannot. In our hands, multiplexing CRISPR guides also facilitates mutant generation by reducing the number of G0 animals to raise. HRM can also be used to cleanup undesired off-target alleles if they occur.

We have demonstrated that HRM can be used to efficiently distinguish particular mutant alleles. In a practical sense, periodically we observe a rare allele, amongst other more common alleles (for example from Table 3 G0#8 the +2 allele) that we want to isolate. Traditional detection methods would prove inefficient or costly (sequencing) at identification of this allele, while with HRM we can identify this allele based on the HRM profile. In future experiments, to characterize the homozygous phenotypes, traditional methods will have difficulty distinguishing homozygous mutant from homozygous wild-type. Even with primer specific design, differentiating a wild-type from mutant allele is difficult to accomplish; however, with HRM this can readily be achieved (as in Fig. 9 wild-type vs. mutant). Along these lines, if we have a choice of multiple out of frame mutations, we often choose the largest deletion, such that it will have the strongest/largest T_m_ change and can be genotyped even using 100 base pair amplicons. In addition, for phenotypic analysis of uncharacterized alleles, HRM is very advantageous in segregating what HRM profiles correlate with a specific phenotype; or more importantly if they do correlate.

HRM does have limitations. Due to the optimal detection with the 100 bp amplicon, HRM will miss larger deletions (>100 bp) that have been described in the literature. However these large deletions are rare and are not necessarily more advantageous that small out of frame deletions. The major drawback to HRM is the setup cost for the equipment required for HRM detection. While traditionally performed in the highly sensitive Lightscanner instrument, a number of other PCR based systems now describe HRM capabilities.

### Future of genome editing and detection

It has been proposed that CRISPRs can be used for biallelic phenotypic analysis in the G0 animals, thereby overcoming the need to breed to the F2 generation [18]. However there are some concerns with this approach: 1) In our experiments we often observe low frequency non-specific phenotypes from CRISPR injections, creating an uncertainty if the phenotypes are due to biallelic targeting or just non-specific non-genetic effects of the nucleases; similar concerns are what has plagued morpholinos; 2) while we do observe greater than 50% germline transmission frequencies, we rarely achieve 100%, suggesting at best this analysis will produce a chimeric phenotype; and 3) with an average out of frame mutation frequency of 2/3, the probability of a both alleles having an out of frame mutation is 4/9, suggesting that <50% of cells or embryos will be biallelic null. We demonstrate an alternative approach that involves intercrossing 2 chimeric G0s, derived from the same CRISPR guide, to generate compound heterozygous or homozygous mutant embryos/fish. While we demonstrate that this approach is effective, to alleviate the concerns for generating homozygous mutations at off-target sites, we believe future approaches can intercross G0s derived from two different guides to the same gene, and avoid these concerns. We believe this approach will also streamline phenotype-genotype correlations by characterizing two separate loci that can only be wild-type or heterozygous. Further, we envision that we can pool multiple CRISPRs to different genes within the same G0s and decipher which phenotypes from G0 intercrosses are due to disruption of specific genes. Extending from this, it may be possible to test complex genetic interactions of two zebrafish paralogs and determine how they influence severity of tissue specificity of phenotypes. Importantly, all phenotype analysis needs to be followed up with careful heterozygous intercross Mendelian-based analysis, but G0 intercrossing does provide a glimpse at potential attractive mutants.

While we have demonstrated the ease in detection of new mutant alleles using HRM, future detection techniques will need to be devised to rapidly identify properly targeted single stranded oligo-directed homologous (ssoHR) recombination alleles [15] as well as engineered deletion alleles. Properly targeted ssoHR alleles could be detected using asymmetrical snap-back oligo based HRM [39]. Due to the ease of HRM, the future bottleneck in genome analysis will move from detection to rapid genotyping of multiple single and complex mutants. For this, balancer chromosomes may be the ideal route. These balancers could be generated through long range genome editing sites on the same chromosome or through loxP oligo based recombineering into nuclease derived sites of the same chromosomes. Alternatively, knock-in of fluorescent marker genes (i.e. cry-DsRed  =  red lens) would also facilitate rapid genotyping. In order for high-throughput and complex genetic analysis to occur, future research will need to address this bottleneck.

### High-through put genome editing

Merging the ease of CRISPR targeting with the efficiency of HRM, high-throughput genome editing can be achieved rapidly and cost effectively. Utilizing a two person team, 30 guides (pools of 3 guides) can be tested and raised per week. Once these G0's are adult then the same two person team can quickly, identify high percentage chimeras through HRM of 12 embryos from G0 x AB. If HRM positive, the remaining F1's embryos will be raised for future allele identification and sperm frozen. High chimeric G0's can then be intercrossed and phenotypes determined. As we have demonstrated HRM genotype of mutants and wild-type embryos can confirm genotype-phenotypes correlations. In addition to analyzing 0–5 dpf phenotypes, the embryos from the G0 intercross will be raised and if the HRM profiles within the embryos do not occur in the adults (∼3 months), then there is a lethality between 5 dpf and the adult stage. We envision with a staggered generation approach that over a 52 week period ∼1500 mutants can be generated and phenotypes analyzed between 0 and 3 months of age.

## Materials and Methods

### Zebrafish lines

All zebrafish work was performed at the facilities of the University of Alabama at Birmingham (UAB) in the Zebrafish Research Facility (ZRF). Adult fish and embryos are maintained as described by Westerfield et al (1995) [40] by the ZRF Animal Resources Program which maintains full AAALAC accreditation and is assured with OLAW. AB wild-type zebrafish were used for RNA injections and controls, and the Esco2 retroviral insertion allele was obtained from Nancy Hopkins and Jacqueline A. Lees [32]. This study was approved by the UAB Institutional Animal Care and Use Committee (IACUC).

### Zinc Finger Nuclease Target Site Selection and Assembly

The ZFN OPEN Pools, pMLM36 (#21871), pB1H2w2-zif268 (#18045) and pH3U3-zif268 (#18046) plasmids were purchased from Addgene. The zinc fingers were designed and selected using the online tool ZiFit Targeter (http://zifit.partners.org/ZiFiT/ChoiceMenu.aspx) [33]. Zinc fingers, targeting p63, p73, ATM, ATR, RB1, and BARD1 were assembled and then screened using a modified bacterial -one- hybrid system similar to that described by Joung et al (2008) [41]. A detailed protocol for assembly and selection of the zinc fingers is available upon request.

### TALEN Target Site Selection and Assembly

TALENs, targeting 23 different genes (including rb1, puma, mdm4, and cyclin g1), were designed and selected using the online tool TAL Effector Nucleotide Targeter 2.0 (https://tale-nt.cac.cornell.edu/node/add/talen) [42]. The Golden Gate TALEN and TAL Effector Kit 2.0 (Catalog number 1000000024) was purchased from Addgene, and TALE repeats were assembled as instructed in the Addgene protocol based on the publication by Cermak et al (2011). Briefly, the TALENs were constructed by combining the desired TAL repeats and performing several cycles of digestion and ligation. These recombined vectors were transformed into Mach1 chemically competent cells to obtain plasmids that could then be digested and ligated into TALEN expression vectors pCS2TAL3DD and pCS2TAL3RR [14]) that contained the Tal constant region, golden gate cloning region and the left or right Fok1 enzyme. Golden gate clones plasmids were used for mRNA synthesis.

### CRISPR Target Site Selection and Assembly

CRISPR guides were designed and selected using the online tool ZiFit Targeter (http://zifit.partners.org/ZiFiT/ChoiceMenu.aspx). The pDR274 (#42250), pT3T3-nCas9n (#46757), and MLM3613 (#42251) plasmids were obtained from Addgene [17], [18]. For CRISPR assembly, we have evolved a number of techniques. Initially, we cloned annealed oligos (containing the guide sequence) into the linearized pDR274, mini-prepped the resulting plasmid, and cut it with the DraI restriction enzyme in order to synthesize RNA. However, to avoid the plasmid prep step and DraI restriction enzyme, we began simply performing colony PCR off of the guide plasmid using the following primers: Forward: TGATTGCAGTCCAGTTACGC and Reverse: GGAGGCTTTTGACTTTCTGCT. The PCR product was used as template for RNA synthesis. We further modified the protocol again by amplifying two approximately 300 bp PCR products (crisprF: acgcccggtagtgatcttat and crisprR: gtgtaaaacgacggccagtt; or crisprF2: gttttagagctagaaatagc and crisprR2: tatagtgagtcgtattagctagcggtgc) off of the pDR274 plasmid using primers that could then be joined together in a second round of PCR with an overlapping reverse primer that contained the desired guide sequence (GCCTTATTTTAACTTGCTATTTCTAGCTCTAAAAC - N^20^ reverse complement of guide-TATAGTGAGTCGTATTAGCTAGCGGTG). This eliminated the cloning step all together and ensured that the guide sequence was present in the 600 bp PCR product. Guide RNA was synthesized off this purified product.

### mRNA Synthesis and Purification

ZFN and TALEN mRNA was transcribed using the mMessagemMachine SP6 kit (Life Technologies). CAS9 mRNA was transcribed from the linearized pT3TS-nCas9n plasmid (Addgene) using the mMessage mMachine T3 kit (Life Technologies). Each RNA was purified using the RNeasy Kit (Qiagen). The CRISPR guide RNA was synthesized using the MegaShortScript T7 Kit (Life Technologies) and purified using the MegaClear Kit (Life Technologies). RNA concentration was quantified using the Nanodrop spectrophotometer.

### Microinjection of Zebrafish Embryos

AB wild-type one-cell stage embryos were collected for microinjection by using a regulated air-pressure micro-injector (Harvard Apparatus, NY, PL1–90). For ZFN and TALEN injections, equal amounts of the Left and Right mRNAs were mixed (starting at 100 ng/ul each and increasing the concentration as needed). For CRISPR/Cas9 injections, 150 ng/ul of Cas9 mRNA and 30 ng/ul of gRNA were mixed together in all experiments. ZFN, TALEN, and CRISPR nucleases were all injected into the yolk of the embryos. These embryos were then used for genomic DNA isolation or raised to obtain G0 adult fish.

### HRM/genomic DNA isolation

#### Genomic DNA

Individual embryos or tail clippings were placed in 100 uL ELB (10 mM Tris pH 8.3, 50 mM KCL, 0.3% Tween 20, 0.3% NP40, 1 mg/ml Prot K) in 96 well plates. Embryos/tail clips were incubated at 55°C for 4 hrs to overnight depending on sample size to generate genomic DNA. To inactivate Proteinase K, plates were incubated at 98°C for 10 minutes.

#### High Resolution Melt (HRM) curve analysis

Primers were designed using Primer3 (http://biotools.umassmed.edu/bioapps/primer3_www.cgi). All primer sets are available upon request. PCR reactions contained 1 ul of LC Green Plus Melting Dye (BioFire Defense), 1 ul of Ex Taq Buffer, 0.8 ul of dNTP Mixture (2.5 mM each), 1 ul of each primer (10 uM), 0.05 ul of Ex Taq (Takara Bio Inc), 1 ul of gDNA, and water up to 10 ul. PCR was performed in an Eppendorf Mastercycler Pro S, using black/white 96 well plates (BioRad cat. No. HSP9665). PCR reaction protocol was 98°C for 30 sec, then 40 cycles of 98°C for 10 sec, 59°C for 20 sec, and 72°C for 15 sec, followed by 95°C for 20 sec and then rapid cooling to 4°C. Melting curves were generated with a Lightscanner HR 96 (Idaho Technology) over a 65–95°C range. Curves were analyzed with LightScanner Instrument and Analysis Software.

### Imaging phenotypes

Embryos were dechorionated by hand using Dumont #5 tweezers and anesthetized using Tricaine. Embryos were positioned in methyl cellulose prior to image acquisition. Brightfield imaging was observed using Nikon AZ100 microscope with 2x objective (0.2 NA) and 2x digital zoom. Z-stacks at 25 µm intervals were taken to encompass the entire embryo. Z-stacks were aligned and focused to create one image. Scale bars denote 500 µm.

## Supporting Information

S1 Table **TALEN targets and primers.** This table contains the gene name, TALEN target sites and primer sequence for HRM analysis. Gene names in red were HRM negative.
(XLSX)

S2 Table **CRISPR targets and primers.** This table contains the gene name, CRISPR target site and primer sequence for HRM analysis. Gene names in red were HRM negative. NT in red in target sites was altered to “G” for targeting.
(XLSX)
