# Correction: High-Throughput Genome Editing and Phenotyping Facilitated by High Resolution Melting Curve Analysis

**DOI:** 10.1371/journal.pone.0117764

**Published:** 2015-02-06

**Authors:** The PLOS ONE Staff

## Notice of Republication

This article was republished on December 30, 2014, to replace Figure 1, which was incorrect. The publisher apologizes for the error.
